# Point-of-Use Water Treatment and Use among Mothers in Malawi

**DOI:** 10.3201/eid1307.070767

**Published:** 2007-07

**Authors:** Lauren J. Stockman, Thea K. Fischer, Michael Deming, Bagrey Ngwira, Cameron Bowie, Nigel Cunliffe, Joseph Bresee, Robert E. Quick

**Affiliations:** * Centers for Disease Control and Prevention, Atlanta, Georgia, USA; † Atlanta Research and Education Foundation, Decatur, Georgia, USA; ‡ University of Malawi College of Medicine, Blantyre, Malawi; § University of Liverpool, Liverpool, UK

**Keywords:** Malawi, diarrhea, social marketing, dispatch

## Abstract

A national household survey was conducted in Malawi to determine awareness and use of a socially marketed water treatment product. In all, 64% of mothers were aware of the product, and 7% were using it. Both poor and rural mothers had lower awareness and use rates. Targeting promotion to rural populations could enhance program effectiveness.

Diarrhea is a leading cause of childhood deaths in the developing world (*1*), where many people rely on drinking water that is contaminated with pathogens. To address this problem, the Centers for Disease Control and Prevention (CDC) and the Pan American Health Organization/World Health Organization developed the Safe Water System (SWS), which consists of water treatment at the point of use with a locally produced, dilute sodium hypochlorite solution, safe water storage, and behavior change techniques such as social marketing (*2*). The SWS has been shown to decrease diarrhea risk by 25%–85% (*3*–*7*) and has been implemented in >25 countries.

In November 2002, an SWS social marketing program was initiated in Malawi to prevent diarrheal illness among children <5 years of age, who were found to have a prevalence of diarrhea of 18% during a 2-week period in the 2000 Malawi Demographic and Health Survey (*8*). The SWS was promoted through radio announcements, flyers, signs on walls and minibuses, and billboards. The disinfectant solution, which was branded as WaterGuard, cost 10 kwacha (approximately $US 0.08) for sufficient solution to treat stored water for 1 month and was sold in small plastic bottles at supermarkets, pharmacies, and by street vendors.

In April 2005, CDC and the University of Malawi conducted a national household survey on healthcare, utilization patterns, and costs of childhood diarrhea and pneumonia in Malawi. We took the opportunity to measure mothers’ awareness, perception, and reported use of WaterGuard.

## The Study

This survey used the “modified segment” design described in the United Nation Children’s Fund’s End-Decade Multiple-Indicator Survey Handbook (*9*), which resulted in an equal-probability sample of 3,000 households in 30 enumeration areas throughout the country. All mothers of children <5 years of age were interviewed. Data were analyzed with SAS-callable SUDAAN 9.0.1 PROC RLOGIST (SAS Institute, Inc., Cary, NC, USA). Multivariate regression models were used to determine predictors of WaterGuard awareness and use. Colinearity and interactions between variables were assessed. To create an indicator of socioeconomic status, household asset factor scores, generated from a principal components analysis from the Malawi 1992 Demographic and Health Survey, were calculated by using the method described by Gwatkin et al. (*10*).

Among 3,000 households included in the survey, 1,787 mothers (or maternal caretakers) were identified, of whom 1,669 (93%) were eligible, having at least 1 child <5 years; all completed the survey. This sample was representative of the population distribution for Malawi, according to the Malawi 2004 Demographic and Health Survey (Table 1).

**Table 1:** Household description of mothers/caretakers interviewed and 2004 Malawi Demographic and Health Survey (DHS) data for comparison

Variable	2005 Household survey		2004 Malawi DHS, %
	No. (%)	95% CI*	
Region			
North	211 (13)	4–34	13
Central	725 (44)	26–63	41
South	716 (43)	26–63	46
Population			
Urban	156 (9)	3–27	18
Rural	1,489 (91)	73–97	82
Latrine			
Traditional pit toilet	1,290 (79)	75–87	79
No facility	289 (18)	13–25	16
Drinking water			
Improved source	1,117 (67)	53–79	64
Unimproved source	539 (32)	21–47	36

*CI, confidence interval.

Among 1,669 mothers, 1,075 (64%; 95% confidence interval [CI] 58–71) had heard of WaterGuard; of these 726 (68%) believed the product was “to make water safe,” 230 (21%) believed the product was “to prevent diarrhea,” and 108 (10%) either did not know or gave another answer. Among the mothers who had heard of WaterGuard, 556 (52%) said they had used it “at some point in the past,” and 124 (12%) reported that they were currently using it. Current users represented 7% (95% CI 4–11) of the total population. Among these, 77 (62%) said that WaterGuard caused “less diarrhea,” or “less illness in the family.” Rates of awareness and use of the product were higher among those living in an urban area than a rural area. Among 432 mothers who had used WaterGuard in the past, but were not using it at the time of the survey, 168 (39%) indicated that they “cannot afford it,” 145 (34%) that it was “currently unavailable,” 12 (3%) that they “don’t like the taste,” and 1 said she didn’t “think it makes water safer.” In all, 106 (25%) gave no reason for no longer using WaterGuard. In a multivariate model, WaterGuard awareness was independently associated with living in an urban area (adjusted odds ratio [aOR] 3.92, p<0.001), being a mother who had attended school (aOR 2.84, p<0.001), having a husband who had attended school (aOR 1.90, p<0.001), and higher wealth quintile (aOR 1.97 p = 0.0003) (Table 2). Current use of WaterGuard was independently associated with living in an urban residence compared to a rural residence (aOR = 2.01, p = 0.0342) (Table 2). The program budget and national WaterGuard sales in Malawi were substantially lower than comparable data from a similar SWS program in Zambia (Figure).

**Table 2:** Univariate and multivariate odds ratios and p value for awareness and current use of WaterGuard*

Predictor variable	Total	No. (%)	Crude OR (95% CI)†‡	Adjusted OR (95% CI)§	p value¶
Have heard of WaterGuard					
Urban population	156	147 (94.2)	9.87 (5.46–17.85)	3.92 (2.26–6.78)	<0.001
Mother attended school	909	713 (78.4)	3.75 (2.59–5.42)	2.84 (2.00–4.05)	<0.001
Husband attended school	1,072	776 (72.4)	2.89 (2.22–3.78)	1.90 (1.45–2.49)	<0.001
Higher wealth quintile	608	471 (77.5)	2.54 (1.69–3.80)	1.97 (1.41–2.74)	0.0003
Region					
Central	723	461 (63.8)	1.15 (0.47–2.80)	NS	
South	708	484 (68.4)	1.41 (0.63–3.14)	NS	
Mother employed	74	59 (79.7)	2.15 (1.03–4.50)	NS	
Improved drinking water	1,106	718 (64.9)	0.95 (0.61–1.47)	NS	
Currently using WaterGuard					
Urban population	144	34 (23.6)	2.86 (1.55–5.28)	2.01 (1.06–3.82)	0.0342
Region					
Central	456	67 (14.7)	3.36 (1.60–7.05)	1.88 (0.93–3.78)	0.1981
South	481	50 (10.4)	2.26 (0.96–5.31)	1.49 (0.60–3.70)	0.1981
Mother employed	58	13 (22.4)	2.22 (1.06–4.67)	1.67 (0.83–3.39)	0.1465
Higher wealth quintile	465	72 (15.5)	1.87 (1.08–3.23)	1.42 (0.84–2.42)	0.1866
Mother attended school	709	91 (12.8)	1.45 (0.69–3.01)	NS	
Husband attended school	771	93 (12.1)	1.22 (0.67–2.21)	NS	
Improved drinking water	712	87 (12.2)	1.22 (0.46–3.21)	NS	

*OR, odds ratio; CI, confidence interval; NS, variable did not meet criterion for remaining in the multivariate model. †OR >1 for region central or region south indicates a higher probability than the north region. ‡OR >1 for higher quintile (4 and 5) indicates a higher probability than those in the lower quintile (1, 2, and 3). §Predictor variables with a p = 0.10 in univariate analysis were included in the multivariate model to adjust for these simultaneously. ¶p value for Wald F statistic for the adjusted OR.

**Figure:**
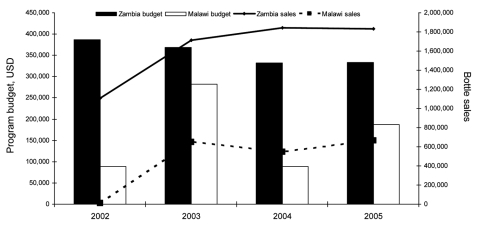
Annual program budget and product sales of Safe Water System programs in Malawi and Zambia, 2002–2005. Year 2005 total population: Zambia, 11,502,010; Malawi, 13,013,926 (www.cia.gov/cia/publications/factbook/geos/za.html). Budget and sales data provided by Population Services International. USD, US dollars.

## Conclusions

This national survey of Malawian mothers found awareness of WaterGuard to be high in a very poor country with limited commercial penetration into rural areas. In addition, over half of mothers who had heard of WaterGuard had tried it, and 12% of these mothers reported currently using the product at the time of the survey. Figures for awareness and past use in the present survey were consistent with an SWS survey conducted in Zambia (*11*), a country that borders Malawi and has a similar poverty and development ranking (*12*). However, reported current use of SWS in Zambia was, at 42%, substantially higher than in Malawi, reflecting substantially higher product sales in Zambia. Unlike the program in Malawi, which has had inconsistent and relatively low levels of funding, the SWS program in Zambia has had stable funding at a higher level and substantially greater sales. If the Malawi SWS program were able to obtain stable funding at higher levels, similar utilization rates to those in Zambia might be attainable.

A substantial gap exists between the percentage of mothers aware of WaterGuard who had tried it and those who were current users at the time of the survey (52% vs. 12%, respectively), which can be considered a dropout rate of 78%. The goal of SWS in Malawi is to increase water quality in an area with limited access to clean water; therefore, sustained use among mothers is as important for long-term health effects as is increasing the initial use of the intervention. The reasons given by mothers who stopped using WaterGuard suggest that cost was a primary barrier to sustained use, especially among rural mothers. More research is needed to better define these and other reasons for discontinuation of water treatment to better inform efforts to increase WaterGuard availability and affordability. The positive perception of WaterGuard among those currently using it, together with the product’s proven ability to disinfect water and prevent diarrhea, justifies continued efforts to market and evaluate the cost effectiveness of WaterGuard in Malawi (*13*).

Overall, findings of this survey support a need to increase WaterGuard promotion and distribution among poorer, less educated, and rural populations. Social marketing programs typically have difficulty reaching rural populations because of inadequate rural commercial infrastructure (*11*). If commercial mechanisms are not sufficient to promote rural use, then alternative, nontraditional approaches should be considered. For example, a program that used trained nurses in a maternal and child health clinic to promote SWS was associated with an increased rate of SWS use in rural Kenya (*14*), and use of motivational interviewing has resulted in higher purchase and usage rates of water disinfectant in Zambia (*15*). Using women’s groups to market and sell products as income-generating activities, may also be efficacious (www.who.int/household_water/resources/freeman.pdf). We recommend that such marketing efforts be targeted to mothers who are least aware of the product and who could benefit the most from safe drinking water, including those who have not attended school, live in a rural area, or are have a lower socioeconomic status.
